# COMBINED KNEE OSTEOARTHRITIS AND DIABETES IS ASSOCIATED WITH REDUCED MUSCLE STRENGTH, PHYSICAL INACTIVITY, AND POORER QUALITY OF LIFE

**DOI:** 10.2340/jrm.v56.39986

**Published:** 2024-09-03

**Authors:** Shi Rui SEOW, Sumaiyah MAT, Jun Jie TEOH, Amyra MOHAMAD YUSUP, Nor Fadilah RAJAB, Intan Safinar ISMAIL, Devinder Kaur Ajit SINGH, Suzana SHAHAR, Maw Pin TAN, Francis BERENBAUM

**Affiliations:** 1 Centre for Healthy Ageing and Wellness (HCARE), Faculty of Health Sciences, Universiti Kebangsaan Malaysia, Kuala Lumpur, Malaysia; 2 Physiotherapy Programme, Faculty of Health Sciences, Universiti Kebangsaan Malaysia, Kuala Lumpur, Malaysia; 3 Laboratory of Natural Products, Institute of Bioscience, Universiti Putra Malaysia, Serdang, Selangor, Malaysia; 4 Ageing and Age-Associated Disorders Research Group, Department of Medicine, Faculty of Medicine, University of Malaya, Kuala Lumpur, Malaysia; 5 Department of Medical Sciences, School of Medical and Health Sciences, Sunway University, Bandar Sunway, Selangor, Malaysia; 6 Rheumatology, Saint-Antoine Hospital, AP-HP, INSERM CSRA, Sorbonne Université, Paris, France

**Keywords:** diabetes mellitus, exercise, mental health, osteoarthritis, knee, physical functional performance, quality of life, social isolation

## Abstract

**Objective:**

This study delves into the intriguing connection between knee osteoarthritis and diabetes in Malaysia. Specifically, the exacerbation of knee osteoarthritis in the presence of diabetes in terms of symptoms, physical performance, physical activity, psychological status, social participation, and quality of life was discussed.

**Design:**

This cross-sectional study recruited adults aged 50 and above by convenient sampling and grouped them into: knee osteoarthritis-diabetes-, knee osteoarthritis+diabetes-, knee osteoarthritis-diabetes+, and knee osteoarthritis+diabetes+.

**Subjects/Patients:**

Of 436 recruited participants, 261 (59.8%) participants reported knee osteoarthritis.

**Methods:**

Handgrip strength, Timed Up and Go test, 6 Meter Walk Test, and 5 Times Sit to Stand Test were measured using standardized procedures. Six questionnaires were administered for the remaining parameters.

**Results:**

Across groups, there were significant differences: 6 Meter Walk Test (*p* = 0.024), Timed Up and Go test (*p* = 0.020), and 5 Times Sit to Stand Test (*p* < 0.001), quality of life (*p* = 0.009), and physical activity (*p* = 0.036). Knee osteoarthritis+diabetes+ was independently associated with reduced handgrip strength, 5 Times Sit to Stand Test, quality of life, and physical inactivity after adjustment. Knee osteoarthritis+diabetes was independently associated with reduced Timed Up and Go test and social isolation.

**Conclusion:**

The findings revealed the diabetic knee osteoarthritis subgroup’s unique physical and psychosocial features of reduced muscle strength and physical inactivity. Future studies should investigate whether managing metabolic factors, and enhancing physical activity and strength exercises, can reduce knee osteoarthritis symptoms and disease severity.

## LAY ABSTRACT

This study characterized diabetes-related knee osteoarthritis by providing insights into the exacerbation of knee osteoarthritis combined with diabetes in terms of knee pain, mobility, daily functionality, emotional well-being, social interaction, and quality of life. Our findings suggested that older adults aged 50 years old and above with both diabetes and knee osteoarthritis shown a significantly different physical and psychosocial profile in comparison with groups with diabetes or knee osteoarthritis alone, and a group without either condition. Living with both diabetes and knee osteoarthritis is linked to an increased likelihood of poorer overall muscle strength, quality of life, and lower physical activity. Those with knee osteoarthritis but no diabetes, however, are more likely to experience social isolation and poor balance. Knowledge of this link between reduced muscle strength and physical inactivity in diabetes-related knee osteoarthritis will allow clinicians to administer individualized approaches in the management of both conditions.

Knee osteoarthritis (KOA) is a common cause of age-related joint diseases leading to joint pain, stiffness, and disability (1). In the Community Orientated Program for the control of Rheumatic Disease (COPCORD) study in Malaysia, 64.8% of Malaysian joint complaints were related to the knee (2), and 1 in 3 Malaysians aged 55 years and above has been reported to have knee pain (3). Previously, only a few KOA risk variables, such as ageing and trauma, were taken into account. However, recent studies have revealed several KOA subgroups, making it a heterogeneous illness. Diabetes mellitus (DM) has, in fact, been identified as a component in the metabolic syndrome phenotype for KOA (4).

Individuals who suffer from both DM and KOA are expected to experience more knee pain compared with individuals without DM (5), while the presence of DM appears to accelerate the development and progression of KOA (6). DM induces systemic chronic inflammation, causing enrichment of advanced glycation end products (AGE) and matrix stiffening, and eventually promotes cartilage degeneration and joint inflammation (7). While previous studies have demonstrated a positive association between DM and KOA (6, 8), only a handful of studies have examined the effect of DM in coexistence on pain and physical performance associated with KOA (5, 9). It has also been suggested that the concomitant presence of both DM and KOA (10) is associated with overall physical activity reduction.

KOA is debilitating in terms of the individual’s physical and psychosocial health (11). Likewise, knee KOA may limit participation in social and certain daily activities (11). Ultimately, the combination of chronic pain, limited mobility, and high disease burden lead to a reduction in quality of life (12, 13). To address the shortage of published studies on the physical and psychosocial effects of the coexistence of KOA and DM, this study aims to explore the level of physical performance, physical activity, psychological status, social participation, quality of life, and knee symptoms among adults aged 50 years old and above in the Klang Valley with and without the presence of KOA and DM. The study findings are expected to inform clinical assessment by healthcare practitioners to facilitate individualized management of individuals with comorbid KOA and DM.

## METHODS

### Study population

This cross-sectional study was conducted on community-dwelling adults aged 50 years and above in the Klang Valley and Selangor through convenience sampling between December 2021 and May 2023. This study was approved by the Universiti Kebangsaan Malaysia Ethics Committee (reference number: JEP-2022-001). Study participants were provided with explanations of research objectives and informed consent was obtained before data collection. Institutionalized older adults or those suffering from major psychological impairment and type 1 diabetes were excluded.

### Data collection

Following recruitment, questionnaires were administered with the assistance of trained researchers, to obtain data on sociodemographic information, self-reported medical history, knee pain, psychological status, leisure-time physical activity, social participation, and quality of life. Anthropometry measurements and physical performance were obtained using a standard procedures.

### Case definitions

The presence of KOA was defined with self-reported physician diagnosed KOA or those in fulfilment of the American College of Rheumatology (ACR) clinical examination criteria (14). Diabetes mellitus was defined as the presence of fasting hyperglycaemia with a serum fasting glucose in excess of 7.00 mmol/L (15) or the presence of self-reported physician diagnosed DM.

### Study outcomes

*Anthropometric measurement.* The height and bodyweight of participants were measured using a height stadiometer (SECA^TM^ 220, Hamburg, Germany) and calibrated weighing scale (SECA^TM^ 769, Hamburg, Germany) respectively. Body Mass Index (BMI) was calculated using the formula: weight [kg] / height² [m²]. Body fat percentage was measured by a body composition analyser (Inbody 270, Cheonan, Chungcheongnam-do).

*Knee osteoarthritis outcome from participants’ perspectives.* The Knee Injury and Osteoarthritis Outcome Score (KOOS) assesses knee pain severity and its associated problems. The tool evaluates both short-term and long-term consequences of knee KOA. It comprises 42 items in 5 separately scored domains: pain, other symptoms, function in daily living (ADL), function in sport and recreation (Sport), and knee-related quality of life (QOL) (16).

### Physical performance and physical activity

*Handgrip strength.* Handgrip strength (HGS) is used for the measurement of overall strength, function, and bone mineral density (17). Participants were instructed to sit with the shoulder adducted and neutrally rotated, with the elbow flexed at 90 degrees. Participants were told not to perform any rapid wrenching or jerking motion throughout the test. Handgrip strength was measured 3 times for each hand using Jamar Dynamometer and the maximum strength from any 1 of the 3 measurements was taken as the HGS (kg) for each hand. Selected cut-offs were based on the Asian Working Group of Sarcopenia (AWGS) consensus criteria, with measurements in excess of 28 kg and 18 kg for men and women, respectively, considered normal (18).

*Timed Up and Go test.* The Timed Up and Go test (TUG) is used to assess the risk of fall, functional mobility, dynamic balance, and lower limb strength (19). Participants should be in a fully seated position on a standard chair with arms resting on the armrests and feet positioned flat on the ground. The time taken to stand up on the command “GO”, walk 3 m at normal pace, then make a U-turn, walk back to the chair, and sit down, was recorded. The test was performed 3 times with the lowest time recorded taken as the final result. A cut-off point of up to 8 s and below was considered normal (20).

*Six Meter Walk Test.* The Six Meter Walk Test (6MWT) assesses gait speed and physical function (21). Participants were instructed to walk at a comfortable speed along a 6-m walkway with markers at 2 m from each end of the walkway to indicate start and finish of measurement area. The timer started as participants crossed the 2-m mark and stopped at the 8-m line. The test was performed once, and the velocity (m/s) was calculated and recorded. The gait speed cut-off point used for the community-dwelling older adults was 1.13 m/s (22).

*Five Times Sit to Stand Test.* The Five Times Sit to Stand Test (5STST) was conducted to measure functional lower limb muscle strength and dynamic balance (23, 24). Participants were instructed to stand up and sit down 5 times quickly but safely. The time started at “GO” and ended when participants completed the 5^th^ repetition. Participants were instructed to stand up completely between each repetition. The lowest time obtained from 3 attempts was taken as the result. Times in excess of 12.8 s were considered as high risk of falls (25).

*Physical activity.* The International Physical Activity Questionnaire (IPAQ) consists of 4 domains, directed to assess the intensity level of physical activity by enquiring on frequencies (days per week) and duration (time per day) of physical activity in the past 7 days. Scores lower than 3000/week were considered physical inactivity (26). The relevance of physical inactivity in the presence of knee osteoarthritis with and without diabetes was then determined.

### Psychological status

The 21-item Depression, Anxiety and Stress Scale (DASS-21) comprises 7 items in each of 3 domains, measured using a 4-point Likert scale: the minimum score of zero indicates “does not apply to me at all” and the maximum score of 3 indicates “applies to me all or most of the time”, establishing a score range of 0 to 21 for each domain. A higher score would indicate a higher level of anxiety, stress, or depression, with cut-off points below 14, 7, and 9 respectively considered normal (27).

### Social participation and network

The Keele Assessment of Participation (KAP) reflects participation in 11 life aspects in the past 4 weeks. The minimum score of “0” indicated no participant restriction while score of 1 to 11 indicated participation restriction in at least 1 activity (28). The 6-item Lubben Social Network Scale (LSNS-6) measures the presence and frequency of social contact with friends and family members. The final total score ranges from 0 to 30, and a score of 12 and lower indicates high risk of social isolation (29).

### Quality of life

Quality of life (QOL) was assessed with the 12-item Control, Autonomy, Self-realization and Pleasure questionnaire (CASP-12). Each item was scored on a 4-point Likert-type scale. With the range of total scores from 12 to 48, higher CASP total scores indicate higher QOL. Within this study, a cut-off score of above 37 was taken to indicate higher QOL and scores (30).

### Statistical analysis

Data analyses were conducted using SPSS Version 20 (IBM Corp, Armonk, NY, USA). The sample population was divided into 4 groups: (*i*) KOA-DM-, (*ii*) KOA+DM-, (*iii*) KOA-DM+, (*iv*) KOA+DM+. Participant characteristics were presented as number (percentage) for categorical variables and median (interquartile range) for non-normal continuous variables. The Kruskal–Wallis test was applied to test for significant difference between the 4 groups, as well as 2-group comparisons for those with and without KOA. Bonferroni adjustment was employed where appropriate to reduce Type I error. Multiple binary logistics regression using dichotomized outcomes was conducted using KOA and DM to show their independent effect, as well as the 4 groups to compare effects between different KOA and DM presence combination, as independent variables with adjustment for potential confounders. The selection of variables to be included relied on statistical analysis and clinical relevance with consideration for potential multicollinearity. Odds ratios (OR) with 95% confidence intervals (CI) were reported. A *p*-value below 0.05 would denote statistical significance.

## RESULTS

### Participants’ characteristics

Data were available from 434 participants, of whom 27.9%, 38.7%, 12.0%, and 21.4% were included as KOA-DM-, KOA+DM-, KOA-DM+, and KOA+DM+ respectively. Table I provides a summary of characteristics of participants within the above 4 groups. Differences existed in age, body fat percentage, BMI, hypertension, hyperlipidaemia, and chronic kidney disease between groups (*p* < 0.05). By comparing the 2 groups with KOA, there were no KOA-related symptom differences despite the presence of diabetes based on the scores in the 5 KOOS domains (Table I).

**Table I:** Baseline characteristics of participants (*n* = 434)

Characteristics	*n*	KOA-DM–	KOA+DM–	KOA-DM+	KOA+DM+	*p*-value
Number (%)	434	121 (27.9)	168 (38.7)	52 (12.0)	93 (21.4)	
Age, median (IQR)	434	65.0 (7.0)	65.5 (7.0)	68.5 (9.0)	66.0 (9.0)	0.018*^‡¶^
Marital status, single, *n* (%)	434	36 (29.8)	58 (34.5)	18 (34.6)	32 (34.4)	0.828
Gender, female, *n* (%)	434	78 (64.5)	120 (71.4)	27 (51.9)	64 (68.8)	0.064
Education level, *n* (%)	434					
Not schooled		2 (1.7)	5 (3.0)	5 (9.6)	8 (8.6)	0.159
Primary education		23 (19.0)	29 (17.3)	10 (19.2)	17 (18.3)	
Secondary education		62 (51.2)	76 (45.2)	26 (50.0)	40 (43.0)	
Tertiary education		34 (28.1)	58 (34.5)	11 (21.2)	28 (30.1)	
Race, *n* (%)	434					
Malay		88 (72.7)	106 (63.1)	32 (61.5)	67 (72.0)	0.144
Chinese		28 (23.1)	50 (29.8)	14 (26.9)	15 (16.1)	
Indian		4 (3.3)	11 (6.5)	6 (11.5)	9 (9.7)	
Other races		1 (0.8)	1 (0.6)	0 (0)	2 (2.2)	
Not living alone, *n* (%)	434	114 (94.2)	149 (88.7)	48 (92.3)	83 (89.2)	0.393
Body fat percentage, median (IQR)	401	36.2 (14.5)	38.3 (13.0)	37.3 (13.2)	40.2 (9.7)	0.022*†
Body mass index, median (IQR)	434	25.6 (6.0)	26.7 (7.2)	26.9 (6.3)	28.8 (6.1)	< 0.001*†^§^
Comorbidities						
Hypertension, *n* (%)	434	45 (37.2)	65 (38.7)	38 (73.1)	67 (72.0)	< 0.001*
Hyperlipidaemia, *n* (%)	434	49 (40.5)	84 (50.0)	37 (71.2)	69 (74.2)	< 0.001*
Heart disease, *n* (%)	434	10 (8.3)	20 (11.9)	4 (7.7)	13 (14.0)	0.473
Chronic kidney disease, *n* (%)	434	0 (0.0)	2 (1.2)	5 (9.6)	6 (6.5)	0.001*
KOOS, median (IQR)						
Symptoms	145	–	82.14 (21.43)	–	78.57 (25.00)	0.451
Pain	143		83.33 (19.45)		80.56 (16.67)	0.208
Activities of daily living	142		8.82 (18.38)		13.24 (16.18)	0.147
Sport	142		35.00 (55.00)		45.00 (55.00)	0.085
Quality of life	142		37.50 (37.50)		37.50 (31.25)	0.293

*P*-values were obtained with ANOVA test for the variables in the table, except for Age, Body Fat Percentage, Body Mass Index, and KOOS subdomains that were analysed with the Kruskal–Wallis test or Mann–Whitney *U* test, and the comorbidities that were analysed with χ^2^ between the 4 groups.

† Indicates significance at *p*-value < 0.05 in pairwise comparison of KOA-DM- and KOA+DM+.

‡ Indicates significance at *p*-value < 0.05 in pairwise comparison of KOA+DM+ and KOA-DM+.

§ Indicates significance at *p*-value < 0.05 in pairwise comparison of KOA+DM- and KOA+DM+.

¶ Indicates significance at *p*-value < 0.05 in pairwise comparison of KOA-DM- and KOA-DM+.

KOA: knee osteoarthritis; DM: diabetes mellitus; IQR: interquartile range; KOOS: Knee injury and Osteoarthritis Outcome Score; *n*: number of cases; SD: standard deviation. Asterisk* indicates significance at *p*-value < 0.05. KOA-DM- indicates absence of both KOA and DM, KOA+DM- indicates KOA presence with absence of DM, KOA-DM+ indicates absence of KOA with the presence of DM, KOA+DM+ indicates presence of both KOA and DM.

### Physical and psychosocial status

Timed Up and Go (*p* = 0.020), 5STST (*p* < 0.001), gait speed (*p* = 0.024), QOL (*p* = 0.009), and physical activity (*p* = 0.036) were significantly different between the 4 groups. No statistically significant existed between HGS (*p* = 0.118), social isolation (*p* = 0.126), social participation (*p* = 0.509), psychological status: depression (*p* = 0.268), anxiety (*p* = 0.118), stress (*p* = 0.209), and the 4 DM/KOA categories (Table II).

**Table II:** Median comparison between groups with and without knee osteoarthritis and diabetes mellitus in terms of physical performance, psychological status, physical activity, health-related quality of life, and social participation, analysed with Kruskal–Wallis test

	*n*	KOA-DM-	KOA+DM-	KOA-DM+	KOA+DM+	*p*-value
Number (%)	434	121 (27.9)	168 (38.7)	52 (12.0)	93 (21.4)	
Physical performance, median (IQR)						
Gait speed	411	1.21 (0.34)	1.12 (0.28)	1.11 (0.35)	1.08 (0.34)	0.024*†
Hand grip strength	417	24.00 (11.75)	22.00 (6.00)	24.00 (11.00)	22.00 (10.00)	0.118
Time up and go	418	8.08 (2.41)	8.78 (2.41)	8.66 (3.08)	9.05 (3.66)	0.020*†
5 Times Sit to Stand Test	414	9.56 (3.73)	10.01 (3.81)	10.38 (4.69)	11.59 (4.82)	<0.001*†^§^
Psychological status, DASS-21, median (IQR)						
Depression	370	0.00 (2.00)	0.00 (4.00)	0.00 (6.00)	2.00 (4.00)	0.268
Anxiety	370	0.00 (4.00)	2.00 (4.00)	2.00 (6.00)	2.00 (6.00)	0.118
Stress	370	0.00 (4.00)	2.00 (4.00)	2.00 (6.00)	2.00 (8.00)	0.209
Physical activity, IPAQ MET, median (IQR)	386	704.25 (2,191.50)	453.75 (1,356.75)	645.50 (1,604.25)	371.25 (1,240.80)	0.036*†
Quality of life, CASP-12, median (IQR)	371	44.00 (7.00)	40.50 (9.00)	42.00 (9.00)	40.00 (10.00)	0.009*†^#^
Social participation, median (IQR)						
LSNS-6 score	362	19.00 (8.00)	17.00 (11.00)	17.00 (10.25)	18.00 (7.50)	0.126
KAP score	351	1.00 (3.00)	1.00 (3.00)	2.00 (2.25)	1.00 (3.00)	0.509

*P*-values were obtained with Kruskal–Wallis test for the variables in the table. Asterisk* indicates significance at *p*-value <0.05.

† Indicates significance at p-value < 0.05 in pairwise comparison of KOA-DM- and KOA+DM+.

# Indicates significance at *p*-value < 0.05 in pairwise comparison of KOA-DM- and KOA+DM-.

§ Indicates significance at *p*-value < 0.05 in pairwise comparison of KOA+DM- and KOA+DM+.

^¶^Indicates significance at *p*-value < 0.05 in pairwise comparison of KOA-DM- and KOA-DM+.

KOA-DM- indicates absence of both KOA and DM.

KOA+DM- indicates KOA presence with absence of DM.

KOA-DM+ indicates absence of KOA with the presence of DM.

KOA+DM+ indicates presence of both KOA and DM.

KOA: knee osteoarthritis; DM: diabetes mellitus; IQR: interquartile range; *n*: number of cases; CASP12: 12-item Control, Autonomy, Self-realization and Pleasure questionnaire; DASS-21: 21-item Depression, Anxiety and Stress Scale; IPAQ: International Physical Activity Questionnaires; MET: Metabolic Equivalent Task; KAP: Keele Assessment of Participation; LSNS-6: Lubben Social Network Scale.

### Multiple logistic regression analysis

Table III displays unadjusted and adjusted associations between KOA alone, DM alone, and both KOA and DM with the outcomes measured. KOA was significantly associated with poor gait speed (OR = 1.54, 95% CI = 1.01, 2.35) and social isolation (OR = 2.06, 95% CI = 1.13, 3.78), while DM was significantly associated with poor 5STST (OR = 1.71, 95% CI = 1.03, 2.84). Both KOA (OR = 1.70, 95% CI = 1.03, 2.81) and DM (OR = 1.93, 95% CI = 1.15, 3.24) were significantly associated with lower QoL (Table III).

**Table III:** Individual association between knee osteoarthritis and diabetes mellitus, with physical performance, psychological status, physical activity, health-related quality of life, and social participation, analysed with logistic regression and presented with odds ratio (OR) and 95% confidence intervals (CI)

Factor	Physical performance				Psychological status			PA	QoL	Social participation	
	Poor HGS (Male < 28 kg, Female < 18 kg)	Poor TUG (> 8.00 s)	Poor GS (< 1.13 ms^-1^)	Poor 5TSTS (> 12.80 s)	Has depression risk (> 9)	Has anxiety risk (> 7)	Has stress risk (> 14)	Low PA, IPAQ MET (< 3000)	Poor to moderate QoL, CASP12 score (< 37)	Socially isolated, LSNS-6 score (< 12)	Socially restricted, KAP score (≥1)
Unadjusted model											
Knee osteoarthritis	1.29 (0.82, 2.03)	1.47 (0.98, 2.21)	1.51 (1.01, 2.27)*	1.40 (0.86, 2.28)	0.88 (0.43, 1.82)	1.36 (0.77, 2.39)	1.06 (0.40, 2.80)	1.94 (1.06, 3.56)*	1.53 (0.94, 2.50)	2.05 (1.14, 3.68)*	0.83 (0.53, 1.28)
Diabetes mellitus	1.60 (1.03, 2.50)*	1.41 (0.91, 2.17)	1.61 (1.05, 2.45)*	2.18 (1.36, 3.50)*	1.61 (0.78, 3.34)	1.48 (0.85, 2.58)	0.79 (0.28, 2.27)	2.21 (1.06, 4.60)*	1.74 (1.07, 2.81)*	0.92 (0.52, 1.63)	1.18 (0.75, 1.87)
Adjusted model											
Knee osteoarthritis	1.39 (0.88, 2.21)	1.39 (0.91, 2.11)	1.54 (1.01, 2.35)*	1.33 (0.80, 2.22)	0.84 (0.40, 1.77)	1.36 (0.77, 2.42)	1.02 (0.38, 2.73)	1.85 (1.00, 3.43)	1.70 (1.03, 2.81)*	2.06 (1.13, 3.78)*	0.87 (0.56, 1.36)
Diabetes mellitus	1.45 (0.89, 2.36)	1.20 (0.75, 1.92)	1.33 (0.84, 2.09)	1.71 (1.03, 2.84)*	1.34 (0.62, 2.94)	1.36 (0.75, 2.46)	0.62 (0.20, 1.94)	1.83 (0.84, 3.94)	1.93 (1.15, 3.24)*	0.70 (0.38, 1.31)	1.30 (0.80, 2.14)
n	417	414	402	414	370	370	370	386	371	361	372

Adjusted model: adjusted for age, comorbidities, and body mass index. Knee osteoarthritis and diabetes mellitus were tested as independent variables on each parameter. Asterisk* indicates significance at *p*-value < 0.05.

HGS: handgrip strength; TUG: Timed Up and Go test; GS: gait speed; 5STST: Five Times Sit to Stand Test; PA: physical activity; QOL: quality of life; IPAQ: International Physical Activity Questionnaires; MET: Metabolic Equivalent Task; CASP12: 12-item Control, Autonomy, Self-realization and Pleasure questionnaire; LSNS-6: Lubben Social Network Scale; KAP: Keele Assessment of Participation; *n* = sample size.

To evaluate the synergistic effects of KOA and DM, multiple groups comparison with the KOA-DM- group as the reference category was used to compare the OR of physical performance, physical activity, psychological status, social participation, and restriction and QOL (Table IV). In comparison with the other 2 groups, physical performance, physical activity level, and QOL were significant for the KOA+DM+ group to different extents, except for social isolation and TUG, which were only significantly associated with the KOA+DM- group compared with the KOA-DM- category. The KOA+DM+ group reported significant associations with poor hand grip strength (OR = 2.00, 95% CI = 1.04, 3.82) and 5STST (OR = 2.21, 95% CI = 1.09, 4.49), which were retained after adjusting for confounding factors. Reduced TUG was observed in all 3 groups, but the significance differences observed in the KOA-DM+ (OR = 2.35, 95% CI = 1.14, 4.83) and KOA+DM+ groups (OR = 1.93, 95% CI = 1.09, 3.42) were attenuated after adjustment, suggesting that the potential confounders may account for reduced TUG in groups with DM. Poor gait speed remained significantly associated with the KOA+DM- (OR = 1.68, 95% CI = 1.01, 2.80) and KOA+DM+ groups (OR = 2.01, 95% CI = 1.09, 3.73) after adjustment. Individuals in the KOA+DM+ group (OR = 3.32, 95% CI = 1.59, 6.97) were significantly more likely to experience lower QOL compared with the KOA-DM- group. Poor QOL appeared to be marginally significantly associated with KOA+DM- after adjustment (OR = 1.90, 95% CI = 1.01, 3.58). The KOA+DM+ group remained significantly more likely to be physically inactive compared with those in the KOA-DM- group after adjustment for all potential confounders (OR = 3.99, 95% CI = 1.26, 12.66). Social isolation was reported to be 2.81 times more likely in the KOA+DM- group, but not the KOA-DM+ and KOA+DM+ groups (Table IV).

**Table IV:** Association between knee osteoarthritis and diabetes mellitus, with physical performance, psychological status, physical activity, health-related quality of life, and social participation, analysed with logistic regression and presented with odds ratio (OR) and 95% confidence intervals (CI)

Item	Parameter	*n*	Models	KOA-DM- (reference), OR (95% CI)		
				KOA+DM-	KOA-DM+	KOA+DM+
Physical performance	Poor hand grip strength (Male < 28 kg, Female < 18 kg)	417	Unadjusted model	1.12 (0.64, 1.95)	1.22 (0.57, 2.64)	2.06 (1.13, 3.77)*
			Adjusted model	1.08 (0.61, 1.91)	0.89 (0.39, 2.03)	2.00 (1.04, 3.82)*
	Poor Timed Up and Go (> 8.00 s)	414	Unadjusted model	1.88 (1.15, 3.07)*	2.35 (1.14, 4.83)*	1.93 (1.09, 3.42)*
			Adjusted model	1.73 (1.05, 2.85)*	1.92 (0.90, 4.10)	1.59 (0.86, 2.92)
	Poor Gait Speed (< 1.13 ms^–1^)	402	Unadjusted model	1.80 (1.10, 2.95)*	2.25 (1.13, 4.50)*	2.36 (1.33, 4.19)*
			Adjusted model	1.68 (1.01, 2.80)*	1.58 (0.76, 3.31)	2.01 (1.09, 3.73)*
	Poor 5 Timed Sit to Stand Test (> 12.80 s)	414	Unadjusted model	1.33 (0.70, 2.50)	2.00 (0.90, 4.51)	3.03 (1.56, 5.88)*
			Adjusted model	1.14 (0.59, 2.20)	1.32 (0.55, 3.14)	2.21 (1.09, 4.49)*
Psychological status	Has depression risk (> 9)	370	Unadjusted model	0.76 (0.30, 1.95)	1.31 (0.41, 4.14)	1.42 (0.54, 3.78)
			Adjusted model	0.71 (0.28, 1.84)	1.03 (0.30, 3.51)	1.13 (0.40, 3.19)
	Has anxiety risk (> 7)	370	Unadjusted model	1.30 (0.64, 2.65)	1.38 (0.53, 3.55)	2.01 (0.93, 4.35)
			Adjusted model	1.31 (0.64, 2.68)	1.25 (0.46, 3.44)	1.84 (0.81, 4.18)
	Has stress risk (> 14)	370	Unadjusted model	0.81 (0.26, 2.48)	0.37 (0.43, 3.14)	0.91 (0.25, 3.35)
			Adjusted model	0.75 (0.24, 2.34)	0.26 (0.03, 2.43)	0.68 (0.17, 2.74)
Physical activity	Low physical activity, IPAQ MET (< 3000)	386	Unadjusted model	1.78 (0.89, 3.53)	1.83 (0.69, 4.90)	4.88 (1.59, 14.91)*
			Adjusted model	1.64 (0.81, 3.29)	1.39 (0.49, 3.92)	3.99 (1.26, 12.66)*
QoL	Poor to moderate QoL, CASP12 score (< 37)	371	Unadjusted model	1.77 (0.95, 3.30)	2.23 (0.99, 5.03)	2.69 (1.34, 5.38)*
			Adjusted model	1.90 (1.01, 3.58)*	2.34 (1.00, 5.50)	3.32 (1.59, 6.97)*
Social participation	Socially isolated, LSNS-6 score (< 12)	361	Unadjusted model	3.06 (1.44, 6.48)*	2.12 (0.77, 5.81)	1.95 (0.80, 4.73)
			Adjusted model	2.81 (1.31, 6.03)*	1.37 (0.46, 4.05)	1.50 (0.59, 3.82)
	Socially restricted, KAP score (≥1)	372	Unadjusted model	0.86 (0.51, 1.45)	1.27 (0.59, 2.72)	0.97 (0.52, 1.82)
			Adjusted model	0.91 (0.53, 1.54)	1.42 (0.64, 3.18)	1.13 (0.58, 2.19)

KOA: osteoarthritis; DM: diabetes mellitus; *n*: sample size; QOL: quality of life; IPAQ: International Physical Activity Questionnaires; MET: Metabolic Equivalent Task; CASP12: 12-item Control, Autonomy, Self-realization and Pleasure questionnaire; LSNS-6: Lubben Social Network Scale; KAP: Keele Assessment of Participation. Adjusted model: adjusted for age, comorbidities, and body mass index.

* Indicates significance at *p*-value < 0.05. KOA-DM- indicates absence of both KOA and DM, KOA+DM- indicates KOA presence with absence of DM, KOA-DM+ indicates absence of KOA with the presence of DM, KOA+DM+ indicates presence of both KOA and DM.

## DISCUSSION

Physical and psychosocial profiles differed between those with KOA and DM compared with those with KOA or DM alone, and those with none. Individuals with both DM and KOA had reduced upper limb and overall muscle strength measured with HGS and 5STST, which did not appear reduced in either KOA+DM- or KOA-DM+ groups compared with KOA-DM-. Similarly, those with both KOA and DM were more likely to be physically inactive and experienced poorer QOL, while the reduction in physical activity and QOL in those with only KOA did not withstand statistical adjustment. Conversely, the presence of KOA with and without DM was independently associated with reduced gait speed, and KOA alone was independently associated with social isolation and reduced TUG, with both parameters appearing as non-significant in those with both KOA and DM compared with those with none. These differences in physical and psychosocial profiles identify the KOA-DM subgroup to be associated with poorer upper limb, overall muscle strength, QOL, and physical inactivity compared with the non-DM KOA subgroup, which is associated with higher fall risk and social isolation.

Both DM and KOA represent common comorbidities linked to reduced QOL; it has been anticipated that individuals with both conditions are also likely to experience more diminished QOL (31). Previous studies have identified that the reduction in QOL in those with KOA was associated with obesity and physical activity (32), while another indicated physical activity, falls, psychosocial consequences, sarcopenia, sexual health, and incontinence as health-related QOL determinants in those with KOA (33). In those with DM, QOL has been found to be associated with use of insulin, complications, and comorbidities (34). The parameter estimated for QOL reduction in those with both DM and KOA in the adjusted model appeared significant compared with those with KOA or DM alone, suggesting first that the contributing factors in either condition would have led to the reduction in QOL in those with both conditions, and having both conditions then has an additive effect to further reduce QOL.

Lower HGS was reported in adults with DM as well as KOA (35, 36). However, divergent perspectives are also available (37), with HGS not reduced in individuals with KOA or DM alone, and the reduction in HGS observed only with the coexistence of both conditions. Insulin resistance and oxidative stress in DM are suggested to have progressively damaged hand collagen structure and function, and advanced KOA manifestation, resulting in lower hand grip strength (9). Prior studies evaluating HGS in those with KOA and DM in isolation had not excluded those with the other condition, hence it is possible that the above mechanism also applies to previous studies. HGS is also considered a measure of overall muscle strength, which in turn represents a marker of sarcopenia in combination with muscle mass (18); this is in line with the poorer lower limb strength determined with 5STST in the diabetic KOA subgroup. In addition, the consistent significant associations with poor 5TSTS scores even after adjustment for potential confounders was in accordance with the hypothesis that both DM and KOA lead to reduction in lower limb strength (38). Biomechanical changes and decreased range of motion in KOA, which leads to poor dynamic balance, together with the influence of diabetic neuropathy in DM, have contributed to poorer lower limb strength in the diabetic KOA subgroup (39, 40).

While a previous study suggested that a decline in muscle mass, strength, and lower limb physical performance occurs in older adults with DM, within this study the reduction in TUG in those with DM alone was accounted for by the differences in age, comorbidities, and BMI (41). The former study had not, however, considered KOA status, hence direct comparisons cannot be made with this study. Reduced gait speed was associated only with KOA, which conflicted with previous findings that having both DM and KOA was most likely to be associated with reduced gait speed, followed by KOA or DM alone (42). This could be explained by the difference in the type of test and cut-off scores used to define poor gait speed. Hence, the result of this study suggests that overall impaired gait and balance represents a feature of the non-diabetic KOA subgroup but not the diabetic KOA subgroup.

The reduction in physical activity observed only among those with both KOA and DM could be rationalized by the synergistic effects of the coexisting conditions (5). Engagement in physical activity could be impeded by mobility impairment from KOA and psychological factors in individuals living with diabetes. This is consistent with a previous study, which demonstrated physical activity impairment in the presence of KOA and DM-related distress (43, 44). In fact, physical inactivity represents a shared risk factor for diabetes and KOA (45), suggesting that the mechanisms underlying KOA in those with DM are metabolic in origin. With the synergistic effects of the coexisting conditions, a vicious circle of physical inactivity and worsening disease control could ensue. Conversely, in those with isolated KOA without DM, other mechanisms may predominate in the development of KOA, such as trauma and occupational factors (46).

Individuals with severe KOA are at higher risk of social isolation (11). Within this study, however, social isolation was a feature of the non-diabetic KOA subgroup. In fact, the relationship between social isolation and DM varies between studies (47). As a heterogeneous chronic disease, adults with DM require individualized management by practitioners in routine care. A possible explanation is that adults with both DM and KOA appeared to have more significant KOA symptoms and pain, requiring more caregiver support for medication adherence and healthcare setting visits, thus reducing risk of social isolation (5, 48).

Unlike the differences observed with physical and social parameters, psychological status did not differ with the presence of DM or KOA. The relationship between diabetes and depression has, in fact, not been established, with the presence of contradictory results from pre-existing literature (49, 50). The differences in psychological status between KOA and DM studies may, however, be explained by geographical or tools differences. The overall low median scores measured using DASS-21 obtained within this study suggest a low number of individuals with depression, anxiety, or stress within the study population, limiting the likelihood of detection of psychological status differences with DM and KOA within this study.

The presence of medical comorbidities was determined by self-report of physician diagnoses, which could have led to recall bias. Nevertheless, errors were minimized using validated questionnaires and with short recall periods. Moreover, chronic disease self-reporting has been found to be reliable in population-based studies (51). Another limitation is that we calculated the sample size based on Cohen’s d from the literature (10), to apply in our non-parametric statistical test for comparison across 4 groups instead of 2; we converted the effect size into eta-squared (η²) and then to effect size, *f*. The approximate total calculated sample size is 96 for a power of 80%; this compensated our effect size adaptation from the literature comparing 2 groups. This study investigated physical performance and activity levels, psychological status, quality of life, social participation and knee symptoms in 4 groups of individuals with and without KOA and DM. Due to the exploratory nature of this study, the results should be interpreted with caution. Nevertheless, we applied Bonferroni adjustment for multiple comparisons to reduce Type I errors. Logistic regression analysis on OA and DM was also performed to rule out false positive findings (see Table III). The characteristics associated with the diabetic KOA might be helpful for physicians in the provision of individualized approaches in the management of both conditions. Future research should also consider targeted interventions to address lower muscle strength and physical inactivity in those with diabetic KOA.

In conclusion, unique characteristics identify those with the coexistence of KOA and DM in terms of reduced physical activity and muscle strength. Further impaired gait and balance in diabetic KOA is accounted for by BMI and percentage body fat, suggesting that the reduction in physical performance in diabetics with KOA may be due to the metabolic effects associated with excess adiposity. In comparison, non-diabetic KOA was independently associated with impaired gait and balance and social isolation. Future studies should seek to determine whether metabolic factors management and interventions to enhance physical activity and strengthening exercises could lead to reduction of KOA symptoms and disease severity in individuals with diabetes.
